# Oscillatory Correlates of Habituation: EEG Evidence of Sustained Frontal Theta Activity to Food Cues

**DOI:** 10.3390/s26031001

**Published:** 2026-02-03

**Authors:** Aruna Duraisingam, Daniele Soria, Ramaswamy Palaniappan

**Affiliations:** 1 School of Computing, Architecture, and Emerging Technologies, Ravensbourne University London, London SE10 0EW, UK; a.duraisingam@rave.ac.uk; 2 Artificial Intelligence and Data Analytics Research Group, School of Computing, University of Kent, Canterbury CT2 7NZ, UK; d.soria@kent.ac.uk

**Keywords:** Electroencephalogram (EEG), food-cue processing, frontal theta oscillations, habituation, time-frequency analysis

## Abstract

Understanding how the brain adapts to repeated food-related cues provides insight into attentional and motivational mechanisms that influence eating behaviour. Previous studies using event-related potentials (ERPs) have shown that food cues, particularly high-calorie stimuli, elicit sustained neural responses with repeated exposure. The present study extends this line of inquiry by examining the oscillatory dynamics of within-session habituation using time-frequency analysis of electroencephalographic (EEG) data from 24 healthy adult participants. Repeated presentations of the same high-calorie, low-calorie, and non-food images were shown, and changes in power across the delta, theta, alpha, beta, and gamma bands were analysed using cluster-based permutation testing. The results revealed a significant habituation effect for the non-food image within the theta band at frontal scalp electrode clusters between 110–330 ms, characterised by a progressive reduction in power over time. In contrast, both high and low-calorie food cues maintained more stable oscillatory activity, indicating sustained attentional engagement. Participant-level analyses further suggested that changes in attentional engagement followed a graded pattern rather than clear categorical differences across stimulus types. These findings suggest that neural habituation is modulated by stimulus salience, with high-calorie food images resisting adaptation through persistent theta-band synchronisation at frontal scalp electrodes. Integrating these oscillatory results with prior time-domain evidence highlights a multi-stage attentional process: an early sensory filtering phase reflected in parietal ERPs and a sustained regulatory phase indexed by theta-band activity recorded at frontal scalp electrodes. This study provides novel evidence that time-frequency analysis captures complementary aspects of attentional adaptation that are not visible in traditional ERP measures, offering a richer understanding of how the brain maintains attention to appetitive visual stimuli.

## 1. Introduction

In the modern environment, individuals are constantly surrounded by food-related cues, ranging from advertisements and social media to everyday settings filled with food options. Repeated exposure to such cues leads to habituation, a neurobehavioural process in which physiological, behavioural, or neural responses gradually decrease following repeated exposure to the same stimulus [1,2]. This reduction has been consistently observed across eating-related responses such as salivation, facial muscle activity, and subjective appetite ratings [3,4]. Habituation is considered an adaptive mechanism that reduces the attentional and motivational relevance of familiar food stimuli and serves as a natural regulator of intake. However, it interacts with hedonic reward systems, cognitive control, and physiological feedback mechanisms [1].

Individual differences in the rate and pattern of habituation have been linked to disordered eating behaviours and obesity [1]. Overweight individuals, particularly children, tend to exhibit slower declines in attentional and motivational responses when repeatedly exposed to food cues, which may lead to increased food intake. This slower habituation also results in higher consumption when new or a variety of foods are introduced, reflecting sustained motivation and heightened reward sensitivity [5]. Furthermore, exposure to a variety of foods, rather than repeated presentation of a single item, has been shown to weaken habituation and increase energy intake [1]. Collectively, these findings indicate that variations in attentional and motivational responses to repeated food stimuli play a crucial role in shaping eating behaviour, suggesting that while habituation is not the sole determinant of overeating, it remains a fundamental component of the broader neurocognitive framework governing appetite regulation and food-related decision-making.

In existing research, neurophysiological tools such as electroencephalography (EEG) and magnetoencephalography (MEG) provide precise temporal insights into how the brain processes food-related cues. Event-related potentials (ERPs) are particularly well suited for capturing rapid attentional and motivational shifts evoked by visual food stimuli [2,6]. Early components such as P200 reflect attentional capture, whereas later components, including P300 and the Late Positive Potential (LPP), represent sustained evaluative or motivational processing [2,7]. In our previous time-domain study, we examined these habituation effects using EEG and found that low-calorie and non-food cues demonstrated apparent attenuation in parietal regions within the 170 to 330 ms window, while high-calorie images maintained sustained responses across repetitions [2]. These findings suggest reduced neural habituation and prolonged attention to high-calorie cues, providing a foundation for exploring frequency-domain mechanisms in the present work. Together, these findings highlight that while habituation to repeated food cues is well established at the time-domain level, important aspects of the underlying neural dynamics remain unexplored. One important limitation of previous work is that ERP measures capture only time-locked activity and cannot fully characterise ongoing oscillatory processes involved in sustained attention and habituation.

Time-frequency analysis of EEG and MEG data enables a detailed understanding of how the brain dynamically processes food-related cues. Traditional ERP studies have contributed substantially to this field, but mainly capture phase-locked neural responses averaged across trials, which limits their capacity to explore ongoing neural dynamics [8]. In contrast, time-frequency analysis measures both phase-locked and non-phase-locked oscillations, allowing researchers to examine how power and phase vary across different frequency bands in response to repeated food stimuli. Oscillatory activity reflects rhythmic neural communication across distributed cortical networks and provides a valuable framework for understanding cognitive and emotional operations such as attention, motivation, and reward processing [9,10]. The coordinated involvement of multiple frequency bands, including delta (1 to 4 Hz), theta (4 to 7 Hz), alpha (8 to 13 Hz), beta (13 to 30 Hz), and gamma (above 30 Hz), illustrates a complex neural system that regulates how food cues are perceived, attended to, and evaluated.

Frontal midline theta activity has been widely studied because of its strong link to motivational salience and cognitive control. An increase in frontal theta power is understood to reflect top-down attentional and evaluative processes, indicating activation of motivational networks when individuals view appetitive stimuli [10,11]. Previous research has shown that frontal theta power can remain elevated across repeated presentations of high-calorie food cues, showing slower habituation compared to low-calorie or neutral stimuli [12]. This persistence suggests continued engagement of reward-related prefrontal and limbic regions [13,14,15]. Overall, frontal theta oscillations appear to serve as a neural marker of sustained motivational attention and may help explain why high-calorie foods remain salient even after repeated viewing.

Other frequency bands also contribute to the processing of food cues. A reduction in alpha-band activity (8–13 Hz), typically observed over parietal and occipital regions, reflects increased cortical activation and visual attention [9,16]. This alpha suppression is often stronger when an individual views appetitive foods, and individuals with overweight frequently show even greater suppression, which may reflect heightened attentional responsiveness to food-related stimuli [7]. Beta and gamma oscillations have also been associated with food-cue perception [17]. Beta activity is linked to evaluative and sensorimotor processing, while gamma synchronisation within occipitotemporal regions contributes to feature integration and reward-related encoding of food images [18,19,20]. Although these higher-frequency oscillatory responses occur briefly in the early stages of stimulus processing, they support rapid perceptual and evaluative analysis of food cues before the more sustained motivational engagement reflected in frontal theta activity [13,14]. Taken together, findings across EEG and MEG research suggest that food-cue perception involves multiple interacting oscillatory systems: alpha, beta, and gamma bands support early sensory and attentional processing, whereas frontal theta plays a central role in maintaining sustained motivational attention toward appetitive food cues [15].

Despite these advances, there has been limited work examining how the brain habituates within a single session when the same food image is viewed repeatedly, particularly in the time-frequency domain. Behavioural studies have demonstrated habituation using measures such as salivation and food intake, and fMRI research has provided additional insight into the neural systems involved [15]. However, oscillatory mechanisms underlying within-session habituation remain poorly understood. Our previous EEG time-domain study showed that low-calorie and non-food cues displayed clear reductions in neural response across repetitions, while high-calorie cues continued to elicit strong responses [2]. The present study extends this work by re-analysing the same EEG dataset in the frequency domain, allowing us to track how oscillatory patterns change with repeated exposures and providing a complementary, more detailed view of neural habituation beyond the time-domain ERP findings previously reported.

Therefore, the present study aimed to examine within-session habituation to repeated high-calorie, low-calorie, and non-food images using time-frequency analysis of EEG data. The objective was to determine whether oscillatory activity patterns differ among these categories. It was hypothesised that food images would evoke more substantial and more sustained attentional responses than non-food images, and that high-calorie cues would elicit greater and slower-to-habituate neural engagement than low-calorie cues. By tracking oscillatory power within a session, this study sought to capture the dynamic neural changes associated with habituation and to provide new insights into how attentional salience influences sustained brain activity during repeated food-cue processing.

## 2. Methodology

### 2.1. Participants

The participant recruitment and selection procedures followed the same methodology described in our previous time-domain study [2]. Twenty-six volunteers (13 males and 13 females) were recruited through university mailing lists and noticeboards, aged between 18 and 48 years (mean = 31.38 ± 7.83). Participants’ liking for apple and pizza was assessed using a six-point Likert scale (0 = not at all, 5 = like extremely), where scores of 0–2 were considered negative, and 3–5 were considered positive. Only those who scored 3 or above for both apple and pizza were included (apple liking: mean = 5.01 ± 0.74; pizza liking: mean = 5.41 ± 0.79). The use of a six-point Likert scale was based on Leung’s recommendation for achieving a normal distribution [21]. Participants with neurological or eating disorders, or those taking medication that could affect their mental state, were excluded from the study.

This study investigated habituation to single visual stimuli, focusing on pizza as a high-calorie food, apple as a low-calorie food, and a hammer as a non-food control stimulus. These stimuli were chosen based on their familiarity and popularity, consistent with a YouGov global survey [22]. The Body Mass Index (BMI) of participants ranged from 17.21 to 39.15 kg/m^2^ (mean = 25.9 ± 5.0), with 14 participants (six males, eight females) classified as overweight or obese. Participation was voluntary, with no financial compensation provided. Due to excessive noise in two EEG datasets, one male and one female participant were removed, leaving 24 participants in the final analysis. All experimental procedures were approved by the University of Kent Faculty of Sciences Research Ethics Committee (ethics approval reference: 0471920), and written informed consent was obtained from all participants.

### 2.2. Experimental Design and Procedure

Participants passively viewed three distinct visual stimuli presented on a monitor positioned one meter away. All images were selected in accordance with the guidelines of validated food image databases, such as Food-Pics [23], using neutral backgrounds, controlled lighting, and isolated objects to ensure visual consistency and experimental control for the habituation analysis. The food items were chosen based on their caloric content and pre-screened participant preference to ensure consistent emotional and neural engagement. All three stimuli (apple, hammer, pizza) used in the experiment are provided as a compressed file in the Supplementary Material. Each participant completed nine sessions in a single day, three sessions for each image type (apple, pizza, and hammer). In each session, the same image was presented 30 times, totalling 90 trials per category and 270 trials overall. The session order was randomised to minimise sequence effects. Each image was presented for 4 s, followed by a 2 s inter-stimulus interval, for a 6 s trial duration. EEG data were time-locked to the onset of each image and analysed within the 4 s viewing window to assess within-session habituation effects. Each session lasted approximately 3 min, with short breaks of 3–5 min between sessions to prevent fatigue. The whole experiment, including setup and rest periods, was completed in about 90 min.

**Figure 1:**
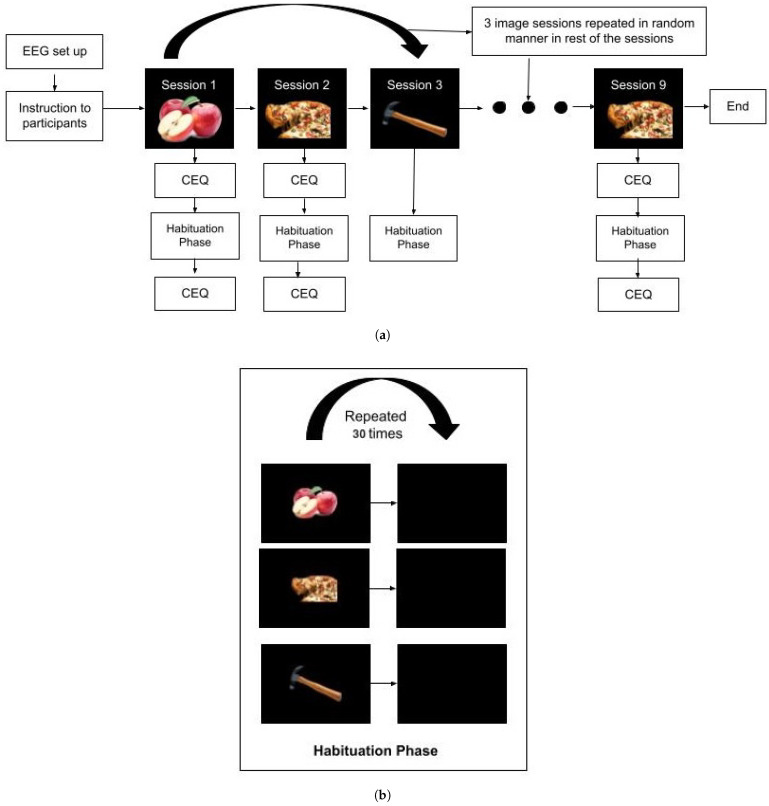
(**a**) Overall habituation experimental design; EEG, Electroencephalogram; CEQ, Craving Experience Questionnaire (participants record their current craving intensity). Sessions 1–3 illustrate the first three habituation blocks, while Session 9 represents a later block shown for illustration; the intervening sessions followed the same structure and were omitted for clarity. (**b**) Habituation phase for each image category: low-calorie, high-calorie, and non-food. All images were displayed for 4 s, followed by a 2 s inter-stimulus interval featuring a blank screen.

At the beginning and end of each session, participants completed the Craving Experience Questionnaire (CEQ) [24] to assess any changes in subjective food craving. Although these measures were collected for reference, no significant differences were observed across sessions or image types, and therefore, CEQ data were not included in the final analysis. Prior to participation, informed consent was obtained, and a screening questionnaire was used to confirm eligibility. To standardise hunger levels, participants were instructed to eat a substantial breakfast and fast for at least three hours before the experimental session, which was consistently conducted at noon across all participants [25]. Upon arrival, they completed the Dutch Eating Behaviour Questionnaire (DEBQ) [26] to assess emotional, external, and restrained eating styles. These measures were collected for potential exploratory analysis but are not discussed further in this paper. Following questionnaire completion, EEG electrodes were fitted, and visual stimuli were presented using the Psychtoolbox (v3.0.16) interface in MATLAB (R2023a). EEG signals were recorded continuously during all sessions, enabling within-session analysis of habituation by tracking oscillatory changes in response to repeated exposures. A schematic representation of the experimental paradigm is provided in Figure 1.

### 2.3. EEG Data Acquisition and Pre-Processing

EEG data were recorded using the StarStim 32-channel wireless system, arranged according to the international 10−10 electrode placement system and sampled at 500 Hz. The CMS (Common Mode Sense) and DRL (Driven Right Leg) electrodes placed on the ear clip served as the reference and ground, respectively. Pre-processing was carried out in MATLAB R2023a using the EEGLAB (v2023.1) [27] and FieldTrip (v20231220) [28] toolboxes. A 1 Hz high-pass filter and 60 Hz low-pass filter were applied to remove slow drift and high-frequency noise. This filter range is commonly recommended for time-frequency EEG analysis as it provides a clearer estimate of oscillatory power changes during cognitive tasks [11,29]. Noisy channels were identified through visual inspection and interpolated. Independent Component Analysis (ICA) was then performed, and artefactual components linked to eye movements, muscle activity, cardiac signals, or line noise with more than 75% probability were identified and removed using the ICLabel classifier, a plugin within the EEGLAB toolbox. The cleaned EEG was segmented from −200 ms to 1000 ms relative to stimulus onset, with baseline correction applied using the −200 to 0 ms pre-stimulus window.

**Figure 2:**
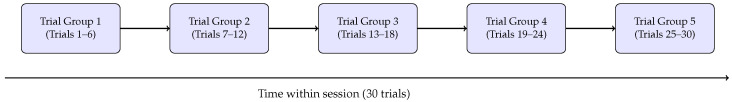
Trial grouping schema used for time–frequency analysis. Each session (e.g., repeated presentation of the same image) was divided into five consecutive trial groups, each consisting of six sequential trials (e.g., Trial Group 1: Trials 1–6). These groups were used to examine within-session neural habituation dynamics.

Time-frequency analysis was carried out using complex Morlet wavelets with a width of five cycles per frequency, providing a balance between temporal and frequency resolution. Power values were computed across the delta, theta, alpha, beta, and gamma bands and normalised to the pre-stimulus baseline. To assess within-session habituation, trials were grouped during data analysis into five consecutive trial sets of six trials each based on their temporal order: Trial Group 1 (Trials 1–6), Trial Group 2 (Trials 7–12), and so on. This grouping was used to track how brain oscillatory activity changed with repeated exposure to the same image while maintaining high data quality for time-frequency analysis. The grouping was applied only during data analysis and was not part of the experimental design. The trial-grouping approach is shown in Figure 2. Statistical comparisons between trial groups were carried out using cluster-based permutation testing to identify meaningful changes in brain activity across time, frequency, and scalp electrodes. The spatial effects reported in this study reflect patterns observed at the level of scalp electrodes that formed significant clusters in the analysis. Because source localisation was not performed, all spatial interpretations are limited to electrode-level distributions rather than specific brain regions.

#### Statistical Analysis

A cluster-based permutation test (CBPT) was used to examine changes across trial groups in the time-frequency data. This approach is appropriate for EEG because it takes into account the temporal and spatial dependency of the signal, identifying clusters of activity that change together rather than treating each time-frequency point as an independent comparison [30]. While CBPT does not fully remove the issue of pseudoreplication, it reduces its influence by performing statistical inference at the cluster level [31]. For each stimulus type, trials were sorted into five sequential trial groups and averaged at the participant level, so that the analysis focused on within-subject changes over repeated exposure. These participant-level averages were then entered into the CBPT, preserving the repeated-measures structure of the design. All statistical analyses were carried out in MATLAB (R2023a) using the EEGLAB and FieldTrip toolboxes, following recommended guidelines for time-frequency cluster-based analysis [28,30].

Statistical comparisons were carried out across multiple frequency bands, Delta (1–4 Hz), Theta (4–7 Hz), Alpha (8–14 Hz), Beta (15–30 Hz), and Gamma (30–60 Hz) and over key cortical regions, including parietal (P7, P3, Pz, P4, P8), frontal (Fp1, Fp2, F7, F3, Fz, F4, F8), central (C3, Cz, C4), temporal (T7, T8), and occipital (O1, Oz, O2) sites. The goal was to identify significant main effects and interactions in time-frequency power associated with repeated visual exposure to high-calorie, low-calorie, and non-food images.

A regression-based approach was implemented using the *ft_statfun_depsamplesregrT* function in FieldTrip to test the hypothesised linear decrease in neural power across repeated exposures (Trial Group 1 > Trial Group 2 > Trial Group 3 > Trial Group 4 > Trial Group 5). Monte Carlo randomisation with 1000 permutations was employed to construct a null distribution of cluster-level t-statistics. Clusters exceeding the 2.5th or 97.5th percentile of this null distribution were considered statistically significant at a two-tailed α=0.05. This non-parametric framework avoids strong distributional assumptions and effectively corrects for multiple comparisons across time, frequency, and electrode dimensions.

This analytical approach allowed us to evaluate within-session habituation while maintaining the repeated-measures structure of the data. It is particularly suited for detecting distributed changes in oscillatory power and provides a robust framework for identifying how attentional processing evolves during repeated visual exposure.

## 3. Results

### 3.1. Behavioural Measures

The behavioural questionnaires were used to describe the participant sample and to check whether eating style or craving changed during the experiment. Normalised DEBQ scores showed similar levels of restrained, emotional, and external eating across participants (restraint: 2.49 ± 0.73; emotional: 2.66 ± 0.64; external: 2.93 ± 0.42). A Friedman test indicated no statistically significant differences between eating styles (χ^2^(2) = 4.37, *p*-value = 0.11), and this was supported by a non-significant trend in the repeated-measures ANOVA (F(2,46) = 2.86, *p*-value = 0.066). As these measures did not differ meaningfully across participants, they were not included in subsequent EEG analyses. Also, subjective craving was measured at the beginning and end of each experimental session, using only food images. For both apple and pizza sessions, craving scores remained stable across the session, with no significant changes from start to end (apple: W = 85.5, *p*-value = 0.183; pizza: W = 160.5, *p*-value = 0.764). These results indicate that repeated exposure to the same food image did not alter self-reported craving during the experimental session. Overall, the behavioural measures confirmed that eating style and subjective craving remained stable throughout the experiment, indicating that the observed EEG changes are unlikely to be explained by behavioural shifts and instead reflect neural responses to repeated visual exposure.

### 3.2. Neurophysiological Measures

**Figure 3:**
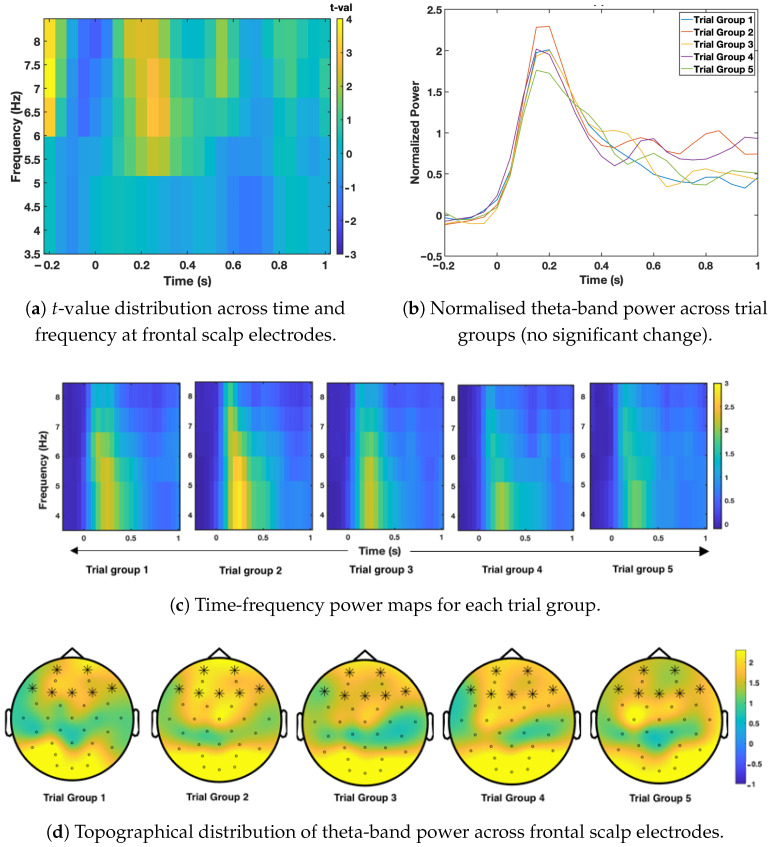
Time-frequency results for the apple condition. (**a**) shows the *t*-value distribution, (**b**) shows theta-band power across trial groups, (**c**) illustrates changes in spectral power over time for each group, and (**d**) shows the spatial distribution of theta activity across the scalp. No statistically significant clusters were identified for this condition (all *p*-values > 0.05).

**Figure 4:**
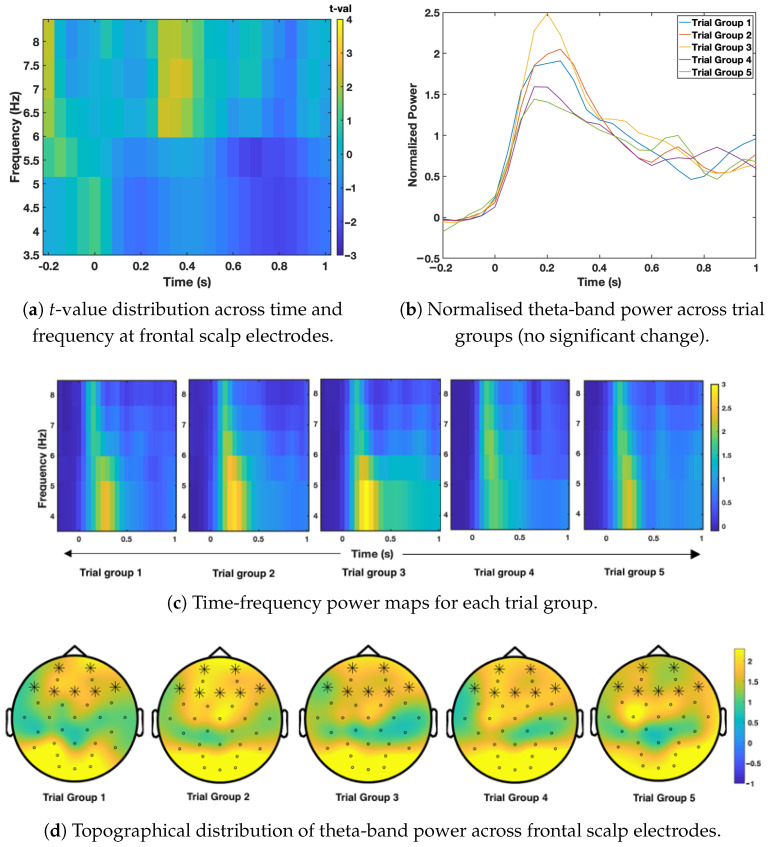
Time-frequency results for the pizza condition. (**a**) shows changes in *t*-values over time at frontal scalp electrodes. (**b**) shows normalised theta-band (4–7 Hz) power averaged across frontal electrodes for each trial group, with no significant changes observed across repetitions (all *p*-values > 0.05). (**c**) presents the time–frequency representation for each group, and (**d**) shows the distribution of theta activity across the frontal scalp. No statistically significant clusters were identified for this condition.

**Figure 5:**
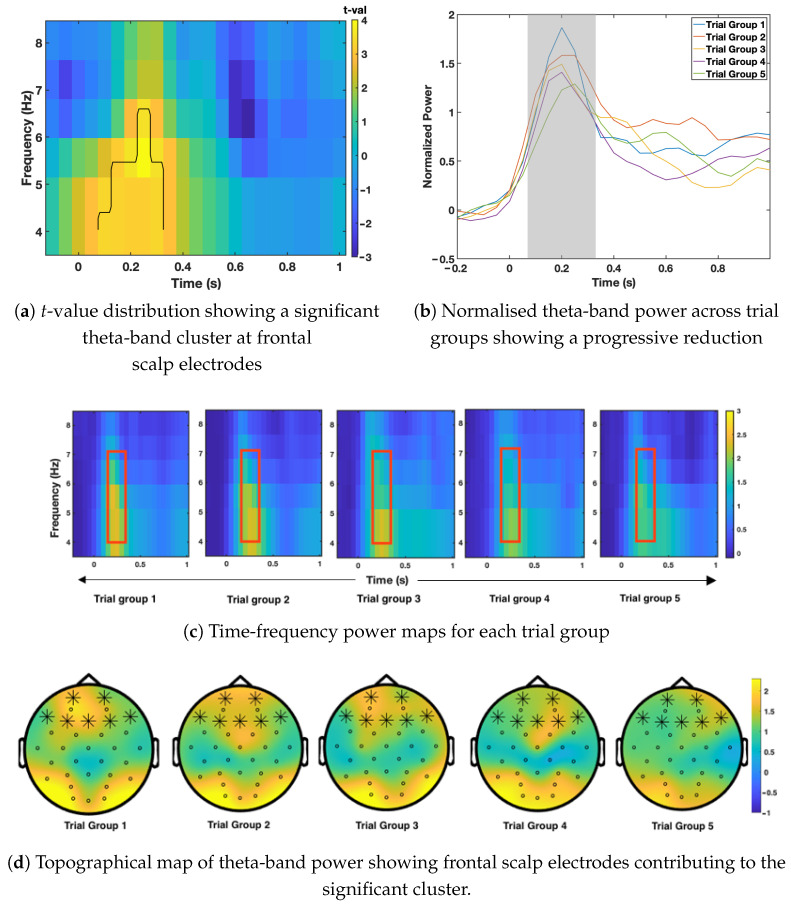
Time-frequency results for the hammer condition. (**a**) reveals a significant theta-band cluster in the *t*-value distribution at frontal scalp electrodes (4–7 Hz, 110–330 ms). (**b**) shows a progressive reduction in normalised theta-band power across trial groups. (**c**) illustrates the time–frequency pattern for each group, and (**d**) highlights the scalp electrodes contributing to the significant cluster. Statistical testing confirmed a significant habituation effect (p=0.011 *). * Asterisk indicates statistical significance (p<0.05, cluster-based permutation test).

Time-frequency EEG analysis was used to examine how brain oscillatory activity changed as the same image was shown repeatedly. Cluster-based permutation testing was applied to identify meaningful changes in oscillatory power across time, frequency, and scalp electrodes for the apple, pizza, and hammer conditions. The results are presented in Figure 3, Figure 4 and Figure 5, following the order of image presentation.

Figure 3 shows the time-frequency results for the low-calorie food image (apple). No significant habituation effects were found in any frequency band or electrode cluster (all *p*-values > 0.05). Although small visual differences were observed across trial groups, these changes did not reach statistical significance. The topographical map also showed no consistent pattern of power reduction, indicating that oscillatory activity remained stable across repeated presentations of the apple image.

Figure 4 presents the results for the high-calorie food image (pizza). As in the apple condition, no significant habituation effects were observed across any frequency band or scalp region. The time-frequency plots showed stable oscillatory patterns across trial groups, and the topographical map confirmed the absence of a clear spatial pattern of power reduction. These findings suggest that neural engagement with the high-calorie image remained consistent throughout the session.

In contrast, Figure 5 shows a clear habituation effect for the non-food image (hammer). A significant reduction in theta-band power (4–7 Hz) was observed at frontal scalp electrodes between 110 and 330 ms after stimulus onset. Theta power was strongest during the earliest trials and gradually decreased across subsequent trial groups (*p*-value = 0.011), indicating a reliable habituation pattern. The topographical map highlights frontal scalp electrodes as the main contributors to this effect, confirming that habituation was spatially specific rather than widespread across the scalp. No other frequency bands showed significant changes, suggesting that the effect was limited to frontal theta activity.

**Figure 6:**
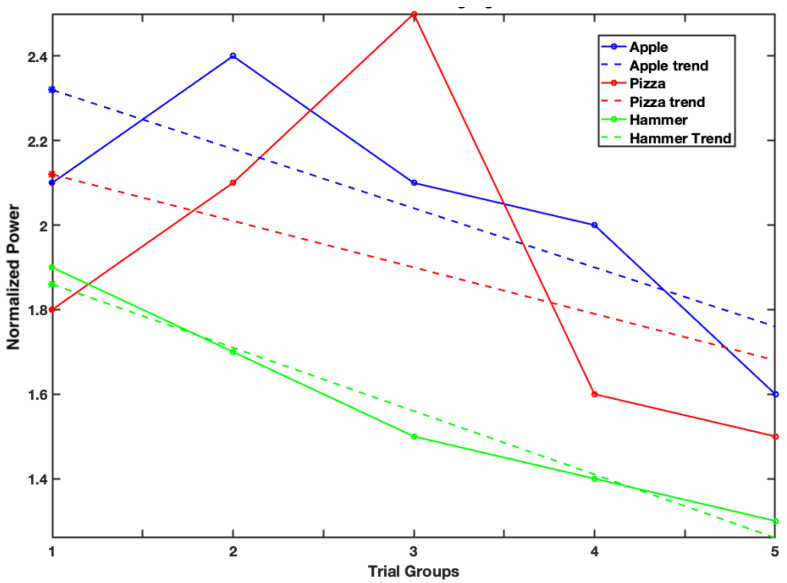
Mean frontal theta (4–7 Hz) power across trial groups for apple, pizza, and hammer conditions. Lines represent group means and illustrate overall habituation trends. Statistical significance was assessed using cluster-based permutation testing at the participant level, which accounts for inter-participant variability.

Figure 6 shows the overall pattern of mean theta-band power across trial groups for the apple (blue), pizza (red), and hammer (green) conditions. For each condition, peak theta-band power was extracted within each trial group, and slopes were calculated to describe changes across successive trial groups. Table 1 summarises the slopes, along with the mean and standard deviation of theta power changes across trial groups, providing an estimate of within-session habituation for each stimulus category. The slopes show a gradual reduction in theta power across repetitions for all conditions, with more negative slopes for the non-food (hammer) and low-calorie (apple) images compared with the high-calorie (pizza) image. To examine differences in habituation rate across conditions, the slopes were compared using a repeated-measures ANOVA. This analysis showed a nominal main effect of condition (F(2,46) = 2.43, uncorrected *p*-value = 0.029), which did not remain significant after Greenhouse–Geisser correction (*p*-value = 0.107). Bonferroni-corrected post-hoc comparisons showed no significant pairwise differences between conditions (all *p*-value > 0.26). Descriptively, pizza images showed a positive mean slope (mean = 0.051), indicating sustained theta-band activity across repetitions, while apple images showed little change (mean = 0.001) and hammer images showed a small negative slope (mean = −0.002), consistent with habituation. The large standard deviations across all conditions indicate considerable variation in habituation rates across individuals. Overall, these findings suggest a graded pattern of habituation rather than clear categorical differences, with food cues showing greater resistance to attenuation than non-food stimuli.

## 4. Discussion

This study is the first to examine within-session habituation to repeated visual food and non-food stimuli using time-frequency analysis of EEG data. By decomposing EEG activity into oscillatory components across both time and frequency, the analysis provided a dynamic view of how attentional processes evolve during repeated exposure to stimuli with different levels of salience. Overall, the results showed that habituation differed systematically across stimulus types, with non-food stimuli showing the fastest attenuation, low-calorie foods showing intermediate habituation, and high-calorie foods showing the slowest reduction in neural response. These findings indicate that habituation is not a uniform neural process but depends strongly on the attentional relevance and behavioural significance of the stimulus.

Theta-band activity recorded at frontal scalp electrodes is understood to reflect sustained attentional monitoring and cognitive engagement, rather than early sensory processing [9,10,11,29]. When theta power decreases over repeated presentations, it indicates that the stimulus is no longer receiving the same level of attention. In contrast, stable theta power suggests continued prioritisation of the stimulus. In the present study, the non-food image showed a clear decrease in theta-band power at frontal scalp electrodes, indicating that attention gradually reduced as the image became familiar. This pattern is consistent with the dual-process theory of habituation, which proposes that repeated exposure to low-salience stimuli leads to reduced orienting responses and lower neural effort [32].

In contrast, the high-calorie image did not show a decrease in theta-band power at frontal scalp electrodes across repetitions, suggesting that attention toward this stimulus remained sustained throughout the session. This is consistent with previous research showing that high-calorie and reward-related stimuli are more motivationally significant and therefore continue to attract and hold attention [33,34]. The low-calorie stimulus showed a different pattern. The ERP results demonstrated a clear habituation effect in parietal electrode channels, indicating that early sensory processing quickly adapted to the repeated low-calorie image [2,35]. However, the time-frequency analysis showed that theta-band activity at frontal scalp electrodes did not reduce to the same extent, suggesting that perceptual familiarity did not fully translate into attentional disengagement. This dissociation highlights that early sensory adaptation and sustained attention may follow different time courses.

The combined ERP and time-frequency findings help differentiate between early sensory processing and later attentional control. The significant reduction in P200 amplitude for the low-calorie stimulus suggests that early sensory-attentional filtering can adapt readily to moderately salient stimuli [2,35]. In contrast, the downward trend (although non-significant) in theta-band power recorded at frontal scalp electrodes indicates that higher-level attentional networks were only partially disengaged. This pattern supports the idea that motivational salience slows the disengagement of sustained attention even when early sensory adaptation has occurred.

The rate of habituation also supports this interpretation. The slopes of theta-band power across repeated presentations showed that the non-food image had the steepest reduction, the low-calorie image showed a moderate reduction, and the high-calorie image showed the smallest change, suggesting slower habituation for more salient food cues. At the same time, there was substantial variability across individuals, and statistical testing indicated that these differences were expressed as graded trends rather than clear categorical effects. Although the repeated-measures ANOVA on slopes showed a trend-level effect of condition, this did not remain significant after correction for sphericity, and no pairwise comparisons reached significance. Descriptively, pizza images showed near-zero or slightly positive slopes, indicating sustained attentional engagement across repeated exposures, whereas apple and hammer images showed negative slopes consistent with gradual attenuation. This pattern suggests that stimuli that are less meaningful or less attention-grabbing habituate more quickly, whereas more salient or rewarding stimuli resist habituation. This interpretation is consistent with behavioural evidence showing that high-calorie food cues tend to hold attention and are harder to disengage from, even after repeated viewing [13,14]. In this context, frontal theta activity may reflect ongoing attentional monitoring for motivationally relevant stimuli, contributing to slower habituation rather than rapid disengagement. Although the pairwise difference between high- and low-calorie food conditions was not statistically significant, the overall pattern suggests a gradual hierarchy of habituation, with high-calorie images showing the slowest reduction and low-calorie images showing intermediate attenuation. This pattern should be interpreted cautiously and may require confirmation in larger samples.

When these findings are considered together with our earlier time-domain ERP results [2], they provide a clearer picture of how the brain responds to repeated visual stimuli. The ERP results showed reduced activity in parietal electrode channels for both low-calorie and non-food images, suggesting that early sensory processing became more efficient as these images became familiar. In contrast, the time-frequency analysis showed a reduction in theta-band power at frontal scalp electrodes only for the non-food image, indicating faster attentional disengagement for low-salience stimuli. Together, these findings support a multi-stage model of habituation involving early sensory filtering followed by later attentional regulation. A key advantage of the present time-frequency analysis is that it reveals sustained oscillatory dynamics that are not captured by ERP measures. While ERPs reflect time-locked sensory and attentional responses, oscillatory activity provides insight into ongoing attentional engagement across repeated exposures. In the current study, this approach revealed that theta-band activity at frontal scalp electrodes remained stable in response to high-calorie food cues despite repeated presentation, a pattern not evident from ERP measures alone. This demonstrates that time-frequency analysis offers complementary information by capturing sustained attentional processes underlying resistance to habituation.

These patterns suggest that habituation occurs in multiple stages. Early sensory mechanisms rapidly adapt to predictable or low-salience stimuli, whereas sustained attentional mechanisms, as reflected at frontal scalp electrodes, remain engaged when stimuli are motivationally relevant. Although this study cannot identify the precise neural sources, the combined evidence suggests that habituation involves coordinated changes across sensory and attentional systems to optimise neural efficiency during repeated exposure.

From a methodological perspective, the sample size was modest, and only one image was used for each stimulus category, which limits generalisability. Although stimuli were presented in a standardised format, caloric content cannot be separated from other visual and semantic properties such as texture, complexity, and familiarity, which may also influence attentional responses. Importantly, habituation was assessed by comparing changes over time within the same image, reducing the influence of between-image visual differences. Future studies should include multiple images per category matched for visual features to further minimise this limitation.

Sex differences were not analysed due to limitations in sample size. For female participants, the menstrual cycle was not controlled, and hormonal changes may have influenced neural responses. Future studies should consider sex differences and the menstrual phase when examining habituation. Future research should also include larger and more diverse samples, examine the role of hunger and eating style, and explore habituation in more naturalistic settings. Combining EEG with eye-tracking or source localisation may further clarify the relationship between neural activity and behaviour, and extending the paradigm over longer timescales may reveal how habituation develops beyond a single session.

## 5. Conclusions

The present study examined within-session habituation to repeated visual food and non-food stimuli using time-frequency analysis of EEG data. By assessing oscillatory activity across frequency bands and time windows, this study provides insight into how attentional processing adapts when visual cues of different salience are repeatedly presented. The key finding is that non-food images showed a reliable reduction in theta-band power recorded at frontal scalp electrodes, indicating faster attentional disengagement from neutral cues, whereas food images showed more sustained theta-band activity across repetitions, reflecting continued attentional allocation to salient stimuli. Although participant-level analyses revealed substantial inter-individual variability and condition differences were expressed primarily as graded trends rather than categorical effects, the overall pattern consistently indicated greater resistance to habituation for food cues relative to non-food cues. Combined with earlier ERP findings, these results indicate that habituation involves both early sensory adaptation and later attentional control mechanisms.

The consistent effects observed in the early latency range (110–330 ms) across both time-domain and time-frequency analyses highlight the precise timing of attentional adjustments during repeated stimulus exposure. Methodologically, integrating time-frequency analysis with cluster-based permutation testing provided a robust framework for capturing both transient and sustained neural responses, extending beyond the limitations of standard ERP averaging. Together, these findings demonstrate that habituation reflects selective attentional adaptation rather than simple neural fatigue, and identify theta-band oscillatory activity recorded at frontal scalp electrodes as a sensitive neural marker of attentional habituation to repeated food-related visual cues.

## Figures and Tables

**Table 1:** Habituation-related change in theta-band power across image conditions. Slopes represent the change in peak theta-band power across successive trial groups, and mean slopes indicate the average rate of change across trial groups for each condition.

Image	Slope	Intercept	Mean Slope	SD
Apple	−0.14	2.46	0.0006	0.0922
Pizza	−0.11	2.23	0.0509	0.1058
Hammer	−0.15	2.07	−0.0024	0.0885

## Data Availability

Data was newly acquired for the present research and can be made available after publication upon request.

## Supplementary Materials

The following supporting information can be downloaded at: https://www.mdpi.com/article/10.3390/s26031001/s1, Compressed file containing images used in the study.

## Abbreviations

The following abbreviations are used in this manuscript:

EEG	Electroencephalogram
ERP	Event-Related Potential
CEQ	Craving Experience Questionnaire
ISI	Inter-Stimulus Interval
CMS	Common Mode Sense
DRL	Driven Right Leg

## References

[1] Epstein L.H., Temple J.L., Roemmich J.N., Bouton M.E. (2009). Habituation as a determinant of human food intake. Psychol. Rev..

[2] Duraisingam A., Soria D., Palaniappan R. (2025). Examining the Neurophysiology of Attentional Habituation to Repeated Presentations of Food and Non-Food Visual Stimuli. Algorithms.

[3] Epstein L.H., Paluch R.A. (1997). Habituation of facial muscle responses to repeated food stimuli. Appetite.

[4] Epstein L.H., Rodefer J.S., Wisniewski L., Caggiula A.R. (1992). Habituation and Dishabituation of Human Salivary Response. Physiol. Behav..

[5] Epstein L.H., Robinson J.L., Temple J.L., Roemmich J.N., Marusewski A., Nadbrzuch R. (2008). Sensitization and Habituation of Motivated Behavior in Overweight and Non-Overweight Children. Learn. Motiv..

[6] Meule A., Kübler A., Blechert J. (2013). Time course of electrocortical food-cue responses during cognitive regulation of craving. Front. Psychol..

[7] Biehl S.C., Keil J., Naumann E., Svaldi J. (2020). ERP and oscillatory differences in overweight/obese and normal-weight adolescents in response to food stimuli. J. Eat. Disord..

[8] Makeig S., Debener S., Onton J., Delorme A. (2004). Mining event-related brain dynamics. Trends Cogn. Sci..

[9] Klimesch W. (2012). Alpha-band oscillations, attention, and controlled access to stored information. Trends Cogn. Sci..

[10] Knyazev G.G. (2007). Motivation, emotion, and their inhibitory control mirrored in brain oscillations. Neurosci. Biobehav. Rev..

[11] Cavanagh J.F., Frank M.J. (2014). Frontal theta as a mechanism for cognitive control. Trends Cogn. Sci..

[12] Gibney K.D., Kypriotakis G., Versace F. (2023). Individual differences in late positive potential amplitude and theta power predict cue-induced eating. Addict. Neurosci..

[13] Nijs I.M., Franken I.H., Muris P. (2010). Food-related Stroop interference in obese and normal-weight individuals: Behavioral and electrophysiological indices. Eat. Behav..

[14] Burger K.S., Stice E. (2014). Neural responsivity during soft drink intake, anticipation, and advertisement exposure in habitually consuming youth. Obesity.

[15] Ghobadi-Azbari P., Mahdavifar Khayati R., Ekhtiari H. (2023). Habituation or sensitization of brain response to food cues: Temporal dynamic analysis in an functional magnetic resonance imaging study. Front. Hum. Neurosci..

[16] Foxe J.J., Snyder A.C. (2011). The role of alpha-band brain oscillations as a sensory suppression mechanism during selective attention. Front. Psychol..

[17] Engel A.K., Fries P. (2010). Beta-band oscillations-signalling the status quo?. Curr. Opin. Neurobiol..

[18] Toepel U., Knebel J.F., Hudry J., le Coutre J., Murray M.M. (2012). Gender and weight shape brain dynamics during food viewing. PLoS ONE.

[19] Polanía R., Krajbich I., Grueschow M., Ruff C.C. (2014). Neural oscillations and synchronization differentially support evidence accumulation in perceptual and value-based decision making. Neuron.

[20] Tashiro N., Sugata H., Ikeda T., Matsushita K., Hara M., Kawakami K., Kawakami K., Fujiki M. (2019). Effect of individual food preferences on oscillatory brain activity. Brain Behav..

[21] Leung S.O. (2011). A Comparison of Psychometric Properties and Normality in 4-, 5-, 6-, and 11-Point Likert Scales. J. Soc. Serv. Res..

[22] Smith Y.M. (2019). Italian Cuisine Is World’s Most Popular. https://yougov.co.uk/topics/consumer/articles-reports/2019/03/12/italian-cuisine-worlds-most-popular.

[23] Blechert J., Meule A., Busch N.A., Ohla K. (2014). Food-pics: An image database for experimental research on eating and appetite. Front. Psychol..

[24] May J., Andrade J., Kavanagh D.J., Feeney G.F., Gullo M.J., Statham D.J., Skorka-Brown J., Connolly J.M., Cassimatis M., Young R.M. (2014). The Craving Experience Questionnaire: A Brief, Theory-Based Measure of Consummatory Desire and Craving. Addiction.

[25] Geisler M.W., Polich J. (1992). P300, Food Consumption, and Memory Performance. Psychophysiology.

[26] Wardle J. (1987). Eating Style: A Validation Study of the Dutch Eating Behaviour Questionnaire in Normal Subjects and Women With Eating Disorders. J. Psychosom. Res..

[27] Delorme A., Makeig S. (2004). EEGLAB: An open source toolbox for analysis of single-trial EEG dynamics including independent component analysis. J. Neurosci. Methods.

[28] Oostenveld R., Fries P., Maris E., Schoffelen J.M. (2011). FieldTrip: Open source software for advanced analysis of MEG, EEG, and invasive electrophysiological data. Comput. Intell. Neurosci..

[29] Cohen M.X. (2014). Analyzing Neural Time Series Data: Theory and Practice.

[30] Maris E., Oostenveld R. (2007). Nonparametric Statistical Testing of EEG- and MEG-Data. J. Neurosci. Methods.

[31] Bae G.Y., Luck S.J. (2018). Dissociable decoding of spatial attention and working memory from EEG oscillations and sustained potentials. J. Neurosci..

[32] Groves P.M., Thompson R.F. (1970). Habituation: A Dual-Process Theory. Psychol. Rev..

[33] Schmidt R., Sebert C., Kösling C., Grunwald M., Hilbert A., Hübner C., Schäfer L. (2018). Neuropsychological and Neurophysiological Indicators of General and Food-Specific Impulsivity in Children with Overweight and Obesity: A Pilot Study. Nutrients.

[34] Stoeckel L.E., Weller R.E., Cook III E.W., Twieg D.B., Knowlton R.C., Cox J.E. (2008). Widespread Reward-System Activation in Obese Women in Response to Pictures of High-Calorie Foods. NeuroImage.

[35] Toepel U., Knebel J.F., Hudry J., le Coutre J., Murray M.M. (2009). The brain tracks the energetic value in food images. Neuroimage.

