# Supplementary figures and images for: Oscillatory Correlates of Habituation: EEG Evidence of Sustained Frontal Theta Activity to Food Cues
Source: Sensors (Basel). 2026 Feb 3;26(3):1001. doi: 10.3390/s26031001 (PMC12900131; doi:10.3390/s26031001)

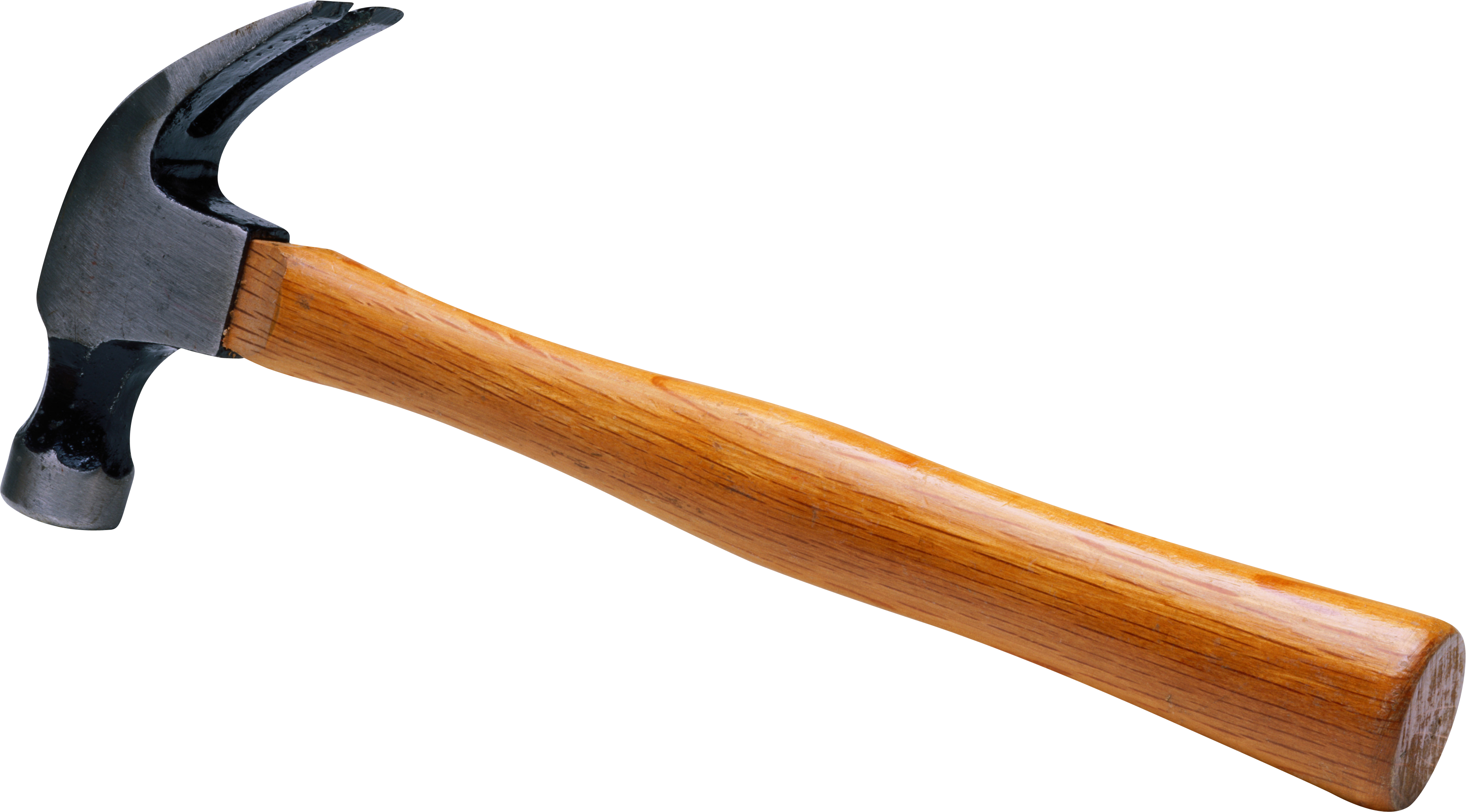

Supplement: Supplementary file 1 [file sensors-26-01001-s001.zip › hammer.png]

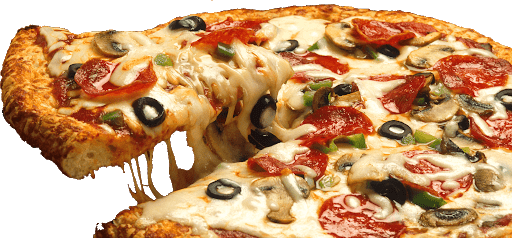

Supplement: Supplementary file 1 [file sensors-26-01001-s001.zip › pizza.png]

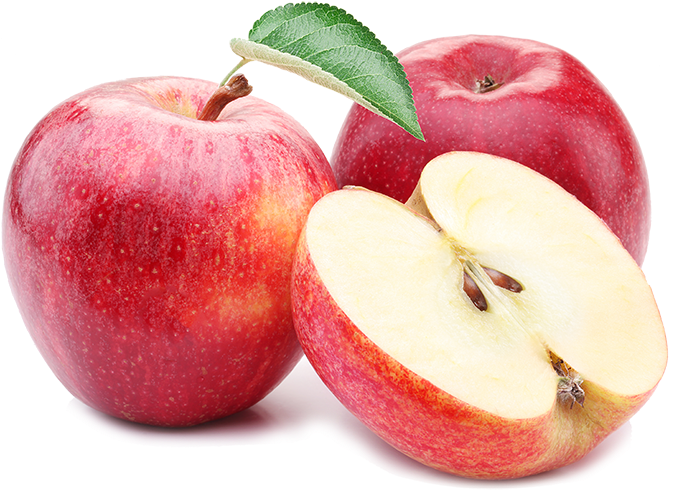

Supplement: Supplementary file 1 [file sensors-26-01001-s001.zip › apple.png]
